# Combination of iKIR missing ligand hypothesis and HLA-B T/T genotypes as predictive biomarkers for recurrent pregnancy loss: a novel algorithmic approach

**DOI:** 10.3389/fimmu.2026.1866398

**Published:** 2026-10-08

**Authors:** Raquel Gil Laborda, Nabil Subhi-Issa, Eduardo de la Fuente Muñoz, María Palacios Ortega, María Guzman-Fulgencio, Angela Villegas Mendiola, MD Mansilla Ruiz, Lydia Pilar-Suarez, Ignacio Cristóbal García, Rafael González Fernández, Silvia Sánchez-Ramón, Miguel Fernández-Arquero

**Affiliations:** 1 Department of Clinical Immunology, Hospital Clínico San Carlos, IdISSC, Madrid, Spain; 2 Immunology, ORL and Ophthalmology Department, Medical Faculty, Universidad Complutense de Madrid, Madrid, Spain; 3 Obstetrics and Gynecology Department, Hospital Clínico San Carlos, Madrid, Spain; 4 Department of Clinical Immunology, Hospital Reina Sofia, Córdoba, Spain

**Keywords:** killer cell immunoglobulin-like receptors (KIR), natural killer cells (NK cells), recurrent implantation failure (RIF), recurrent pregnancy loss (RPL), unexplained recurrent failure (uRF)

## Abstract

**Introduction:**

Natural killer (NK) cell alloreactivity at the maternal–fetal interface is regulated by killer cell immunoglobulin-like receptors (KIR) and CD94/NKG2A receptors. A dimorphism at position −21 of the HLA-B leader peptide (−21M/−21T) biases NK education toward NKG2A (when −21M supplies HLA-E–binding peptides) or toward KIR (when −21T fails to do so). We evaluated whether maternal HLA-B leader genotype and inhibitory KIR (iKIR) missing-ligand (ML) status are associated with unexplained recurrent reproductive failure (uRF).

**Methods:**

Retrospective case–control study comparing recipient HLA-B leader genotype (M+ versus T/T) and iKIR ML across HLA-C groups with outcomes in uRF patients versus healthy controls. 77 women with uRF (43 recurrent pregnancy loss [RPL], 34 recurrent implantation failure [RIF]) and 75 healthy multiparous controls. Maternal KIR, HLA-B and HLA-C genotypes were typed from peripheral blood using Luminex-based SSP/SSO assays.

**Results:**

The centromeric KIR AA haplotype was enriched in uRF versus controls (difference 24%, p=0.0056). iKIR-ML analysis showed higher frequency of KIR2DL2/3 with absent C1 ligands in uRF (p=0.015). The HLA-B −21 analysis revealed increased T/T homozygosity in uRF (p=0.0031). The combination of KIR2DL2/3 (absent C1) with −21 T/T was more prevalent in RPL (p=0.04).

**Discussion:**

HLA-B −21 T/T and iKIR ML (absent C1 for KIR2DL2/3) may aid risk stratification in RPL. Findings warrant validation in larger cohorts.

## Graphical Abstract

**Figure:**
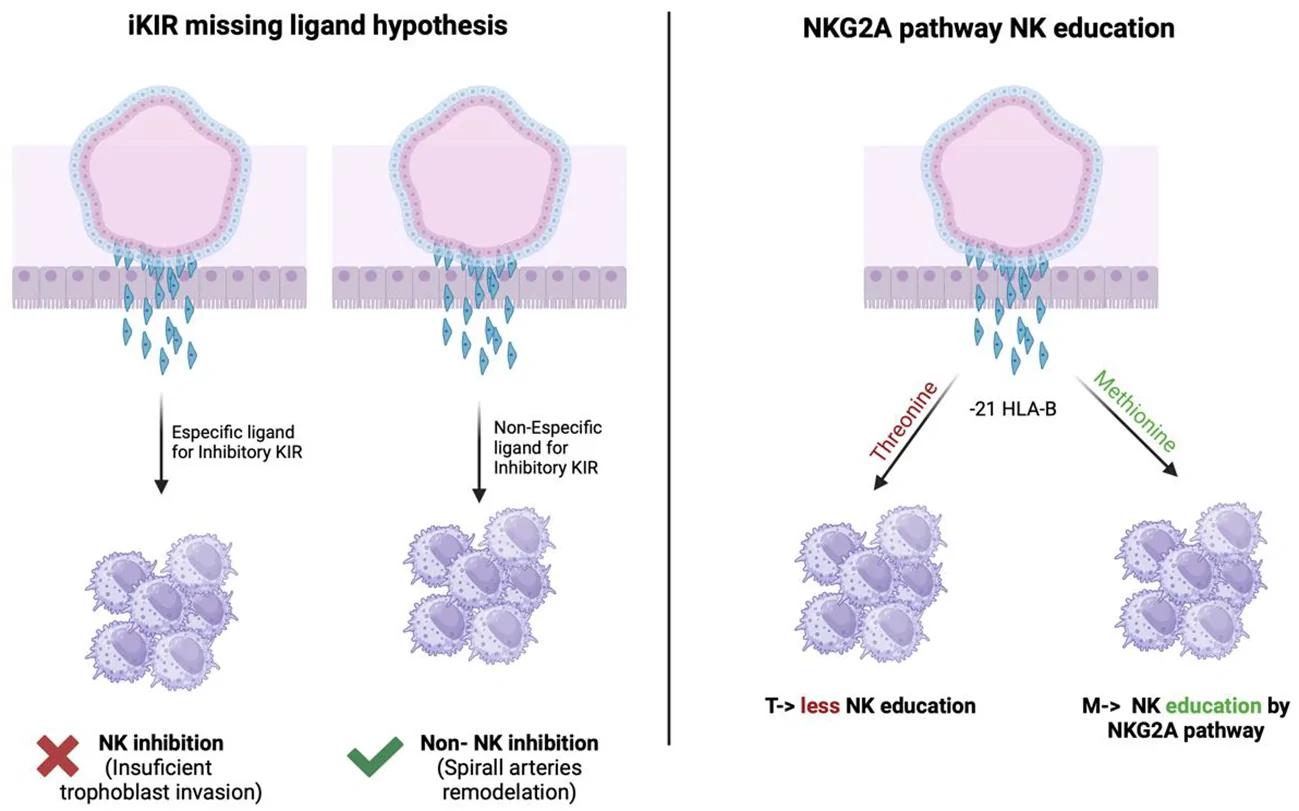

## Introduction

The immune system contributes critically to placentation through interactions between uterine NK (uNK) cells and extravillous trophoblast (EVT) in the decidua (1). Key receptor systems for NK cell function include Killer cell Immunoglobulin-like Receptors (KIR) and CD94/NKG2A (2, 3). KIR receptors recognize classical polymorphic HLA alleles (-A, -B, -C), while NKG2A recognizes the non-classical HLA-E, which requires binding to peptides from HLA-A, -B, or -C for membrane expression (4, 5).

For HLA-E to interact with class I HLA leader peptides, methionine (M) at position 21 is essential (21M). Most HLA-A and HLA-C alleles contain this methionine, while many HLA-B alleles have threonine (T) at this position (21T), leading to ineffective binding (6). Patients may have HLA-B leader genotypes of M/M, M/T, or T/T, with NK cells educated in an M+ context being more activated and cytokine-secreting than those in a T/T context (7–9).

KIR genes, located on chromosome 19q13.4, include seven inhibitory (iKIR) and six activating (aKIR) genes. KIR2DL1 recognizes specific HLA-C allotypes (group C2), while KIR2DL2/3 targets others (group C1). KIR3DL1 interacts with the Bw4 epitope, which is present not only on a subset of HLA-B alleles but also on several HLA-A haplotypes (e.g., A23, A24, A*32), thereby modulating NK cell responses across class I molecules (4, 10–13).

The ‘missing ligand’ hypothesis, originally described in hematopoietic stem cell transplantation, does not refer to ligand downregulation, but to the constitutional absence of the cognate HLA class I ligand for an inhibitory KIR receptor. In this model, donor NK cells expressing an inhibitory iKIR but lacking the corresponding HLA ligand in the host can become alloreactive. Our study adapts this conceptual framework to the decidual setting, evaluating the genetic absence of ligands for key iKIRs. In recurrent pregnancy loss (RPL), lack of KIR ligands could promote NK cell activity, improving tissue remodeling and infection defense. Conversely, certain HLA-B alleles may increase susceptibility to complications by disrupting immune balance (14).

Understanding the interplay between HLA-B dimorphism and KIR ligand availability is essential for grasping the genetic factors influencing health outcomes. While some studies link donor KIR or receptor ligands to transplant success (14, 15), others do not (2).

The role of NK cell-mediated alloreactivity in pregnancy remains uncertain. Additionally, the HLA-B leader genotype may raise preeclampsia risk by reducing HLA-E expression, leading to decreased uNK cell formation and inhibited NK cell function, ultimately affecting spiral artery revascularization (1, 16).

This study aims to analyze the contributions of the HLA-B receptor leader genotype and iKIR ML-mediated NK cells in women with RPL compared to multiparous controls. We hypothesize that both the M+ HLA-B genotype and the presence of iKIR ML will support proper fetal development.

## Material and methods

### Study design

Our study was designed as a retrospective, observational case-control study, firstly we want to confirm the importance of centromeric-telomeric gen KIR study, then we specifically aimed at assessing whether the classification of -21 HLA-B dimorphism and iKIR hypothesis introduce additional clinical significance beyond what is known.

### Subjects

Women with RPL or RIF were consecutively referred to the Reproductive Immunology Unit, Hospital Clínico San Carlos (2021–2024). Within the uRF cohort, 43 fulfilled ESHRE criteria for RPL (≥2 pregnancy losses, including non-visualized) and 34 met criteria for RIF (failure to achieve clinical pregnancy after >3 high-quality embryo transfers or ≥10 embryos transferred in women <40 years). Healthy controls (HC; n=75) were multiparous women with ≥2 uncomplicated pregnancies from Hospital Clínico San Carlos and Hospital Reina Sofía. All participants provided informed consent; the study followed the Declaration of Helsinki and was approved by the local Ethics Committee (FIS PI19/01450). All participants self-reported Caucasian ancestry.

Participants were recruited during routine outpatient evaluation at the Reproductive Immunology Unit. Peripheral blood samples were obtained while all women were non-pregnant. Inclusion criteria for the uRF group comprised women fulfilling ESHRE criteria for recurrent pregnancy loss or recurrent implantation failure. Exclusion criteria included chromosomal abnormalities, uterine malformations, active infection, autoimmune diseases other than antiphospholipid antibody positivity, and known causes of reproductive failure. Healthy controls were multiparous women with two or more uncomplicated pregnancies and no history of infertility, recurrent miscarriage, or placentation disorders and less than 40 years.

### HLA-B, HLA-C and KIR genotyping

DNA was isolated using the MagNA Pure platform (Isolation Kit I – Large Volume, Roche). Genotyping used Immucor Lifecodes eRES SSO kits for HLA-B (Ref 628917), HLA-C (Ref 628921) and KIR (Ref 545110R) on Luminex equipment (Hospital Universitario Clínico San Carlos). HLA-B alleles were annotated for Bw4/Bw6 motifs and grouped by the leader peptide at position −21 as −21TT, −21MM, or −21MT. KIR typing included 14 genes (2DL1, 2DL2, 2DL3, 2DL4, 2DL5, 2DS1, 2DS2, 2DS3, 2DS4, 2DS5, 3DL1, 3DL2, 3DL3, 3DS1) and two pseudogenes (2DP1, 3DP1). HLA-C alleles were dichotomized by residue 80 (C1: Asn; C2: Lys).

Centromeric (Cen) and telomeric (Tel) KIR gene-content motifs were assigned as follows: Cen-A versus Cen-B by presence of 2DL3 versus 2DS2–2DL2; Tel-A versus Tel-B by 3DL1–2DS4 versus 3DS1–2DS1. KIR2DS3, KIR2DS5 and KIR2DL5 were considered part of Cen-B/Tel-B motifs regardless of physical location. Genotype categories were: AA (two Cen-A and two Tel-A), BB (two Tel-B), and Bx (all other combinations). The copy number of KIR2DL1A was inferred from Cen-A dosage as described previously.

Not all samples yielded complete genotyping results for every locus analyzed. Because KIR, HLA-C, and HLA-B −21 typing were performed using independent SSP/SSO assays, a small proportion of samples did not meet the laboratory quality-control criteria for specific markers. These failures included insufficient DNA quantity, ambiguous probe hybridization patterns, or incomplete amplification of leader-sequence regions. For this reason, the number of individuals included in each genetic comparison reflects the subset of samples with valid and high-confidence genotyping for the specific locus or receptor–ligand pair under analysis.

All individuals remained part of the study cohort, and no participants were excluded from the dataset itself. Only marker-specific comparisons show reduced sample sizes when genotyping for that locus was incomplete.

This study expands upon our previously published analysis of centromeric KIR haplotypes (17). While part of the cohort overlaps with the previous report, the current investigation addresses a distinct research question focusing on HLA-B -21 dimorphism, inhibitory KIR missing-ligand interactions, and their combined predictive value. These analyses were not included in the prior publication.

As noted, the absence of specific ligands for killer cell immunoglobulin-like receptors (KIR) on natural killer (NK) cells is critical in contexts like hematopoietic stem cell transplantation (HSCT). In classical NK education models, developing NK cells that encounter self-HLA ligands through inhibitory receptors (such as KIR2DL1 or KIR2DL2/3) become functionally competent, whereas NK cells lacking such interactions remain hyporesponsive (18, 19). Therefore, the absence of inhibitory KIR ligands does not enhance NK education. However, in the decidual microenvironment, uNK cells acquire functions distinct from peripheral NK cells. In this context, reduced engagement of inhibitory KIRs may decrease inhibitory tone and facilitate tissue remodeling rather than cytotoxicity, despite not constituting enhanced education per se. Our interpretation refers to this local modulation of uNK activity, not to classical NK educational licensing.

### Statistical analysis

Demographic and clinical variables were compared using χ² tests, one-way ANOVA or Kruskal–Wallis as appropriate. Logistic regression assessed associations between KIR/HLA-C genotypes and outcomes, reporting odds ratios (OR) with 95% confidence intervals (CI). Stratified analyses (RPL, RIF) were prespecified. Two-sided p<0.05 denoted nominal significance. To control family-wise error where multiple independent genotype-proportion tests were performed, we applied Bonferroni correction across four primary comparisons (C1C1, C1C2, C2C2 frequencies within Cen-AA; and combined KIR–HLA interactions), yielding α=0.0125 (0.05/4). Secondary analyses are reported unadjusted and interpreted cautiously.

To control family-wise error where multiple independent genotype-proportion tests were performed, Bonferroni correction was applied across four predefined primary comparisons, yielding a significance threshold of α=0.0125. Secondary and exploratory analyses are reported using nominal p-values and should be interpreted cautiously.

## Results

### Epidemiological and obstetrical features

We analyzed 77 patients with uRF group were referred to our Reproductive Immunology Unit between 2021 and 2024 for systematic analysis, of which 75 were included in the health control group. As shown in **Table 1**, an analysis of the obstetrical history revealed differences between the healthy control group and the uRF group, thus allowing for further comparative analysis. All of them are Caucasian.

**Table 1:** Clinical and demographical features of the study groups.

Variable	Total (HC)	Positive aPL (uRF)	Negative aPL (uRF)	P value
Patients uRF	n=75	n = 13 (17.3%)	n = 64 (82.66%)	
Aged (años), mean (SD)	40.7 (3.4)	40.3 (3.0)	40.8 (3.5)	.458
BMI (kg/m2), mean (SD)	23.3 (3.8)	24.5 (3.9)	23.1 (3.8)	.130
Smoke n (%)	7 (9.09%)	0 (0.0%)	7 (11.11%)	.336
IVF previous, n (%)	45 (58.44%)	6 (42.85%)	39 (61.9%)	.107
Previous miscarriage n (%)	0 (0%)	6 (42.85%)	24 (38.09%)	.681
Previous miscarriage, median (RIC)	0 (0)	3.3 (2.0;4.6)	2.0 (0.7;3.3)	.615
Children, n (%)	10 (13.3%)	0 (0.0%)	10 (15.62%)	.064
NKs > 13, n (%)	32 (41.0%)	8 (53.3%)	24 (38.1%)	.281

(n)= number, natural killer (NKs), Unexplained reproductive failure (uRF) patients: n=77; Healthy Control (HC): n=75. In vitro fertilization (IVF), antiphospholipids antibodies (aPL). Data are mean±SD or n (%).

### Centromeric AA KIR in uRF versus healthy control group

The first aim was to compare and validate the difference between classic KIR generation and KIR generating haplotypes by separating the centromeric region from the telomeric. As previously observed (20), there is a loss of information when generating classic haplotypes, specifically losing between 10-15% of patients with Cen AA KIR haplotype, in both patients and controls.

Separating centromeric from telomeric KIR regions avoids information loss inherent in classical AA/AB/BB assignments and reduces misclassification of patients as Bx when they are Cen-AA. In our cohort, the Cen-AA genotype was significantly enriched in uRF group (56.41%) versus controls (34.7%; p=0.0075), replicating our previous observations. Conversely, Cen-BB haplotype was reduced in uRF group (5%) versus controls (14%; p=0.04). Classical categorization obscured approximately 16% of Cen-AA cases (diagnosed as AB), supporting separate analysis of Cen and Tel regions. (**Figures 1C, E**, **Supplementary Data 1**).

**Figure 1:**
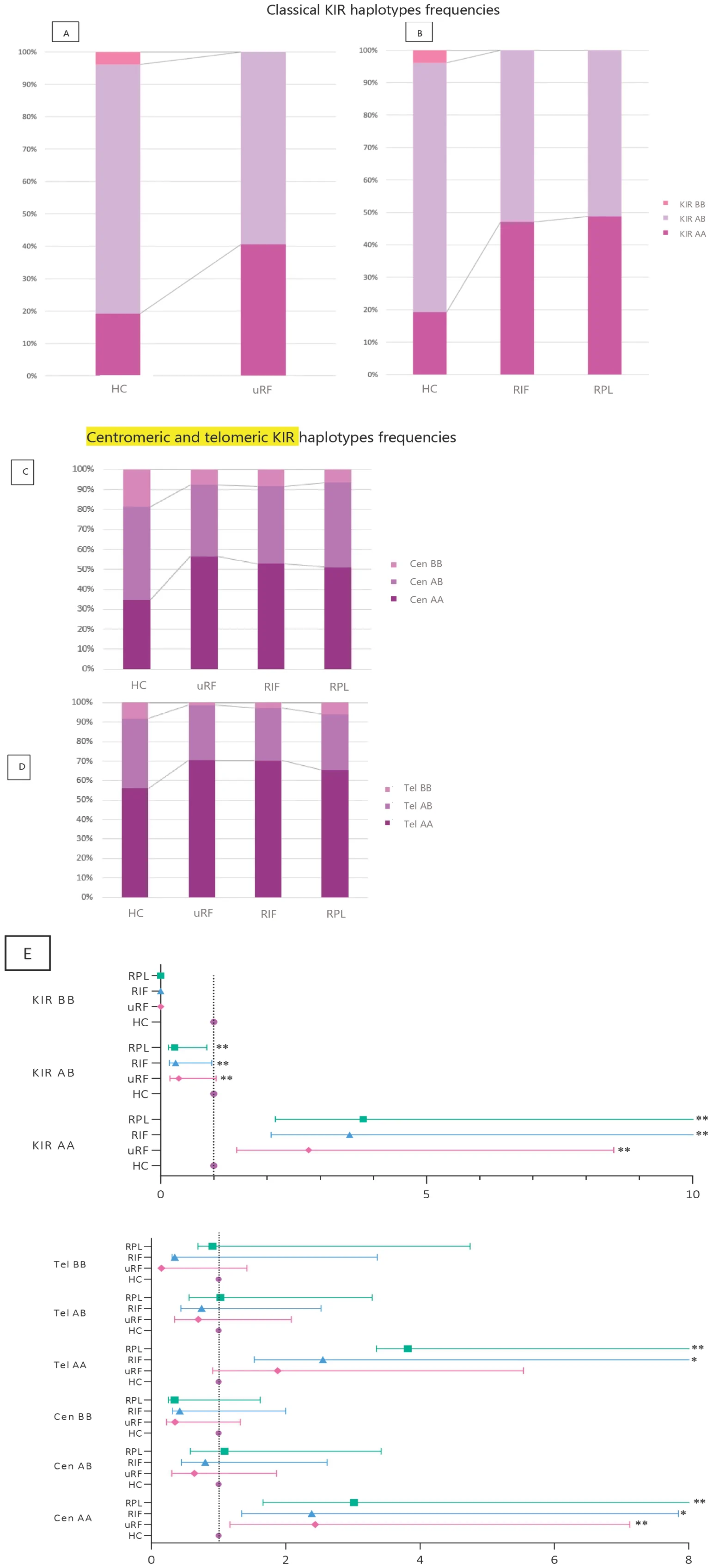
Classical, centromeric and telomeric KIR haplotypes frequencies **(A)** Frequency of classical KIR haplotypes in patients with unexplained recurrent failure (uRF) and the healthy control group (HC). **(B)** Frequency of classical KIR genotypes in patients with recurrent implantation failure (RIF) and recurrent pregnancy loss (RPL) compared to the healthy control group (HCG). **(C)** Frequency of centromeric motif haplotypes of KIR genotypes in patients with with unexplained reproductive failure (uRF), recurrent reproductive failure (RPL) and the healthy control group (HC). **(D)** Frequency of telomeric motif KIR genotypes in patients with unexplained reproductive failure (uRF), recurrent implantation failure (RIF) and recurrent pregnancy loss (RPL) compared to the healthy control group (HCG). **(E)** Representation of Odds Ratios and 95% confidence intervals for the uRF, RPL, and RIF groups compared to the reference population. Data shown. **P<0.01; *P<0.001. HC=75 patients, uRF=77 patients, RPL=43 patients, RIF 34= patients.

Furthermore, if we compare RPL and RIF groups with the HC group, CenAA is present in 58.53% and 55.88% respectively, compared to 34.67%. This also has a significant p-value in both cases: 0.007 in RPLgroup and 0.0393 in RIF group (**Figures 1C, E**, **Supplementary Data 1**).

### HLA-C and KIR centromeric haplotype combination

Given the confirmed loss of information when studying only classical KIR, we conducted an algorithmic analysis associating the centromeric KIR haplotype with the HLA-C haplotype in women. Those with the KIR CenAA haplotype showed a significant increase in homozygosity for C1 in the HC group, with a p-value of 0.0058 (**Figures 2A, E**; **Supplementary Data 2**).

**Figure 2:**
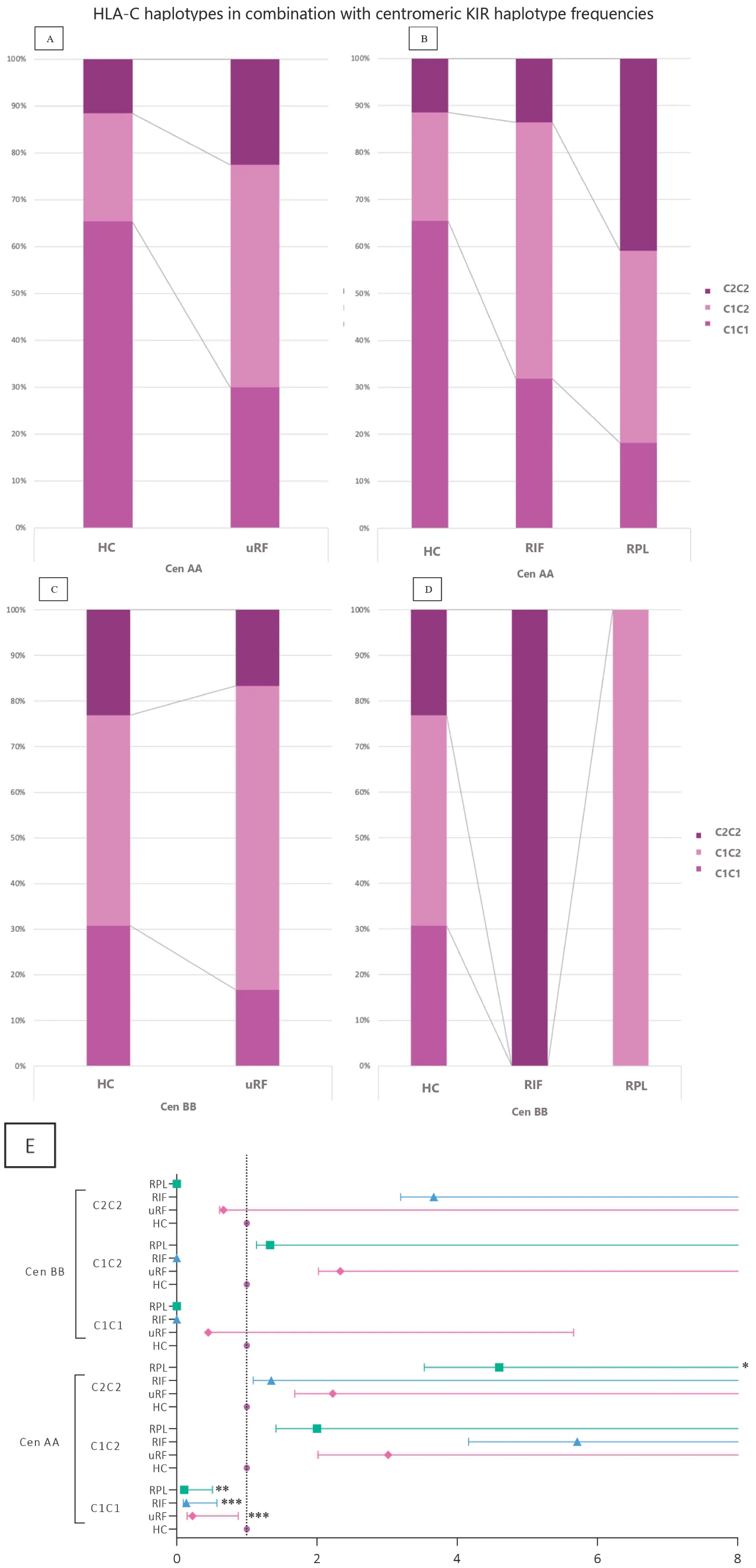
HLA-C haplotypes in combination with centromeric KIR haplotype frequencies. **(A)** Frequency of different HLA-C haplotypes in patients with unexplained recurrent failure (uRF) in combination with KIR cen AA haplotype, and the healthy control group. **(B)** Frequency of different HLA-C haplotypes in patients with recurrent implantation failure (RIF) and recurrent pregnancy loss (RPL) in combination with KIR cen AA and the healthy control group. **(C)** Frequency of different HLA-C haplotypes in patients with unexplained reproductive failure (uRF) in combination with KIR cen BB, and the healthy control group. **(D)** Frequency of different HLA-C haplotypes in patients with recurrent implantation failure (RIF) and recurrent pregnancy loss (RPL) in combination with KIR cen BB and the healthy control group (HC). **(E)** Representation of Odds Ratios and 95% confidence intervals for the RPL, AR, and RIF groups compared to the reference population. Data shown **P<0.01; *P<0.001. HC=75 patients, uRF=77 patients, RPL=43 patients, RIF 34= patients. ***, <0.001.

In examining the heterozygosity of the C haplotype with KIR CenAA haplotype, patients with uRF group exhibited a significant prevalence (47.50%) compared to controls (23.07%), yielding a p-value of 0.049, suggesting that C2 prevalence may indicate a risk of pregnancy loss. While homozygosity for C2 with KIR CenAA haplotype did not show statistical significance, there was an increase in the uRF group (22.50% vs. 11.50%) compared to HC group, although sample size limited confirmation (**Figures 2A. E**; **Supplementary Data 2**).

No significant associations were found with the centromeric BB motif and the three C haplotypes; however, a trend was noted. The prevalence of C1C1 with CenBB in controls may suggest a protective mechanism against RPL, warranting further investigation with larger samples.

The frequency patterns of HLA-C haplotypes combined with KIR CenAA hplotype in the RIF and RPL groups mirrored those in the overall recurrent reproductive failure group, indicating a significant reduction in homozygosity for C1 compared to HC group (p=0.0008 and p=0.0011). Notably, homozygosity for C2 was significant in the RPL group (37.5% vs. 11.5% in HC group, p=0.0404) (**Figures 2B, E**; **Supplementary Data 2**).

Lastly, while the data for HLA-C combined with KIR CenBB haplotype in the uRF group were displayed, no significant results were found due to limited sample size.

### iKIR missing ligand hypothesis in pregnancy

We specifically examined the KIR2DL1 receptor, the most potent inhibitory receptor, in relation to uRF group. In this framework, we observed that complete absence of HLA-C2 for KIR2DL1 was more prevalent in HC group (45.95%) than in uRF group(26.9%; p=0.0187. (**Figures 3A**, **4**; **Supplementary Data 3**).

**Figure 3:**
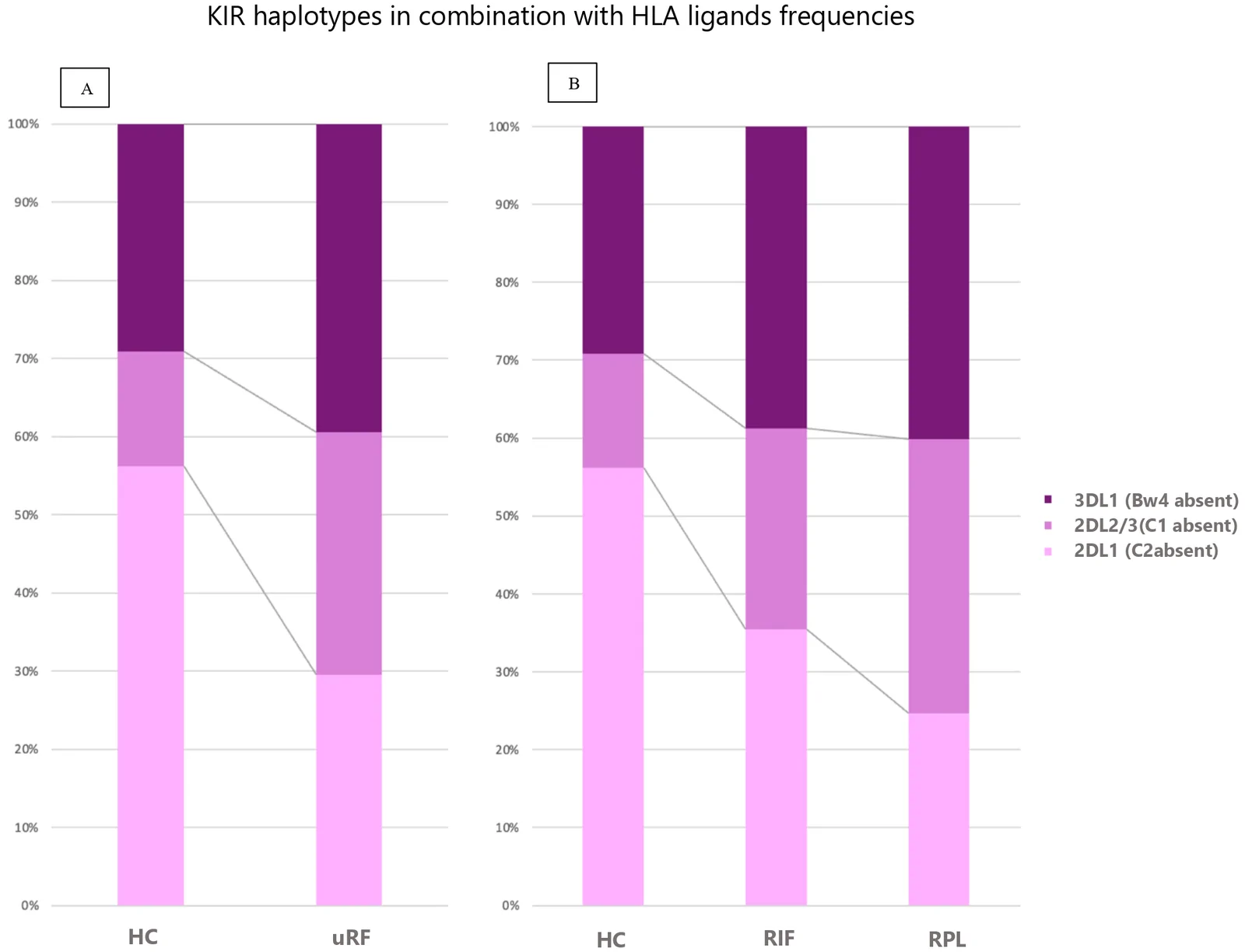
KIR haplotypes in combination with HLA ligands frequencies. **(A)** Frequency of different HLA-C haplotypes in patients with unexplained reproductive failure (uRF) in combination with KIR cen AA haplotype, and the healthy control group. **(B)** Frequency of different HLA-C haplotypes in patients with recurrent implantation failure (RIF) and recurrent pregnancy loss (RPL) in combination with KIR cen AA haplotype and the healthy control group (HC). HC=75 patients, uRF=77 patients, RPL=43 patients, RIF 34= patients.

**Figure 4:**
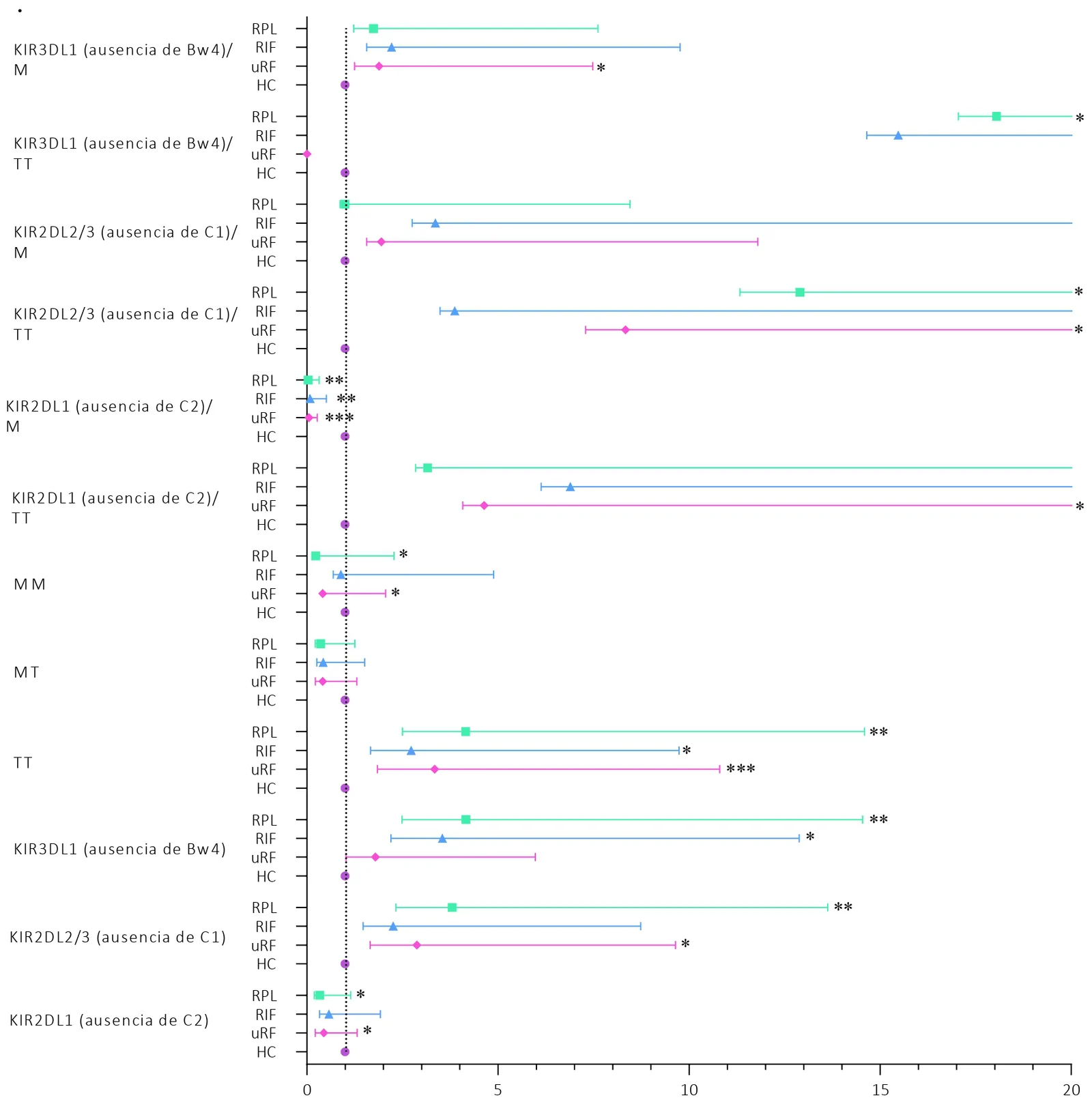
Representation of Odds Ratios and 95% confidence intervals for the unexplained reproductive failure (uRF), recurrent implantation failure (RIF) and recurrent pregnancy loss (RPL) compared to the healthy control group (HC) in dimorphism in HLA-B -21 position and iKIR ML hypothesis. Data shown **P<0.01; *P<0.001. HC=75 patients, uRF=77 patients, RPL=43 patients, RIF 34= patients. ***, <0.001.

When the data were stratified RIF group and RPL group, no significant differences were observed in the RIF group. In contrast, the RPL group showed a significant association with KIR2DL1 (p = 0.0146) (**Figures 3B**, **5**; **Supplementary Data 3**). We also evaluated the receptors KIR2DL2 and KIR2DL3, both of which recognize the HLA-C1 ligand. Notably, the absence of HLA-C1 was significantly higher in the uRF group (28.2%) compared with the HC group (12%, p = 0.015), suggesting a potential protective role for this ligand (**Figures 3A**, **4**; **Supplementary Data 3**).

**Figure 5:**
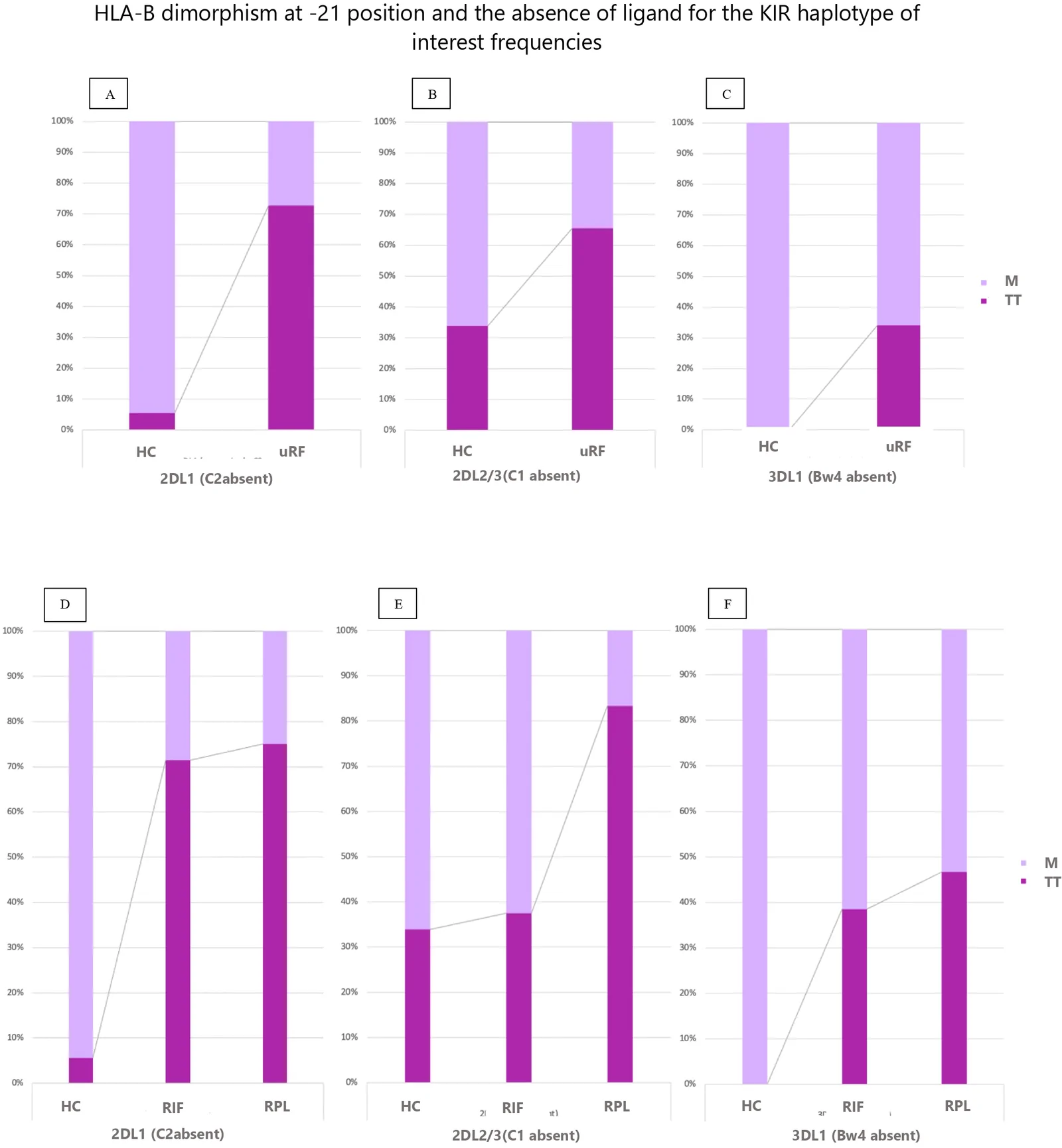
HLA-B dimorphism at -21 position and the absence of ligand for the KIR haplotype of interest frequencies. **(A)** Frequency of the missing ligand hypothesis for KIR2DL1 combined with the HLA-B dimorphism at position -21 in patients with unexplained reproductive failure (uRF) and the healthy control group (HC). **(B)** Frequency of the missing ligand hypothesis for KIR2DL2/3 combined with the HLA-B dimorphism at position -21 in patients with unexplained reproductive failure (uRF) and the healthy control group (HC). **(C)** Frequency of the missing ligand hypothesis for KIR3DL1 combined with the HLA-B dimorphism at position -21 in patients with unexplained reproductive failure (uRF) and the healthy control group (HC). **(D)** Frequency of the missing ligand hypothesis for KIR2DL1 combined with the HLA-B dimorphism at position -21 in patients with recurrent pregnancy loss (RPL) and recurrent implantation failure (RIF) compared to the healthy control group (HC). **(E)** Frequency of the missing ligand hypothesis for KIR2DL2/3 combined with the HLA-B dimorphism at position -21 in patients with recurrent pregnancy loss (RPL) and recurrent implantation failure (RIF) compared to the healthy control group (HC). **(F)** Frequency of the missing ligand hypothesis for KIR3DL1 combined with the HLA-B dimorphism at position -21 in patients with recurrent pregnancy loss (RPL) and recurrent implantation failure (RIF) compared to the healthy control group (HC). M encompasses MM and MT. HC=52 patients, uRF=77 patients, RPL=43 patients, RIF 34= patients.

In the RIF group, no statistically significant differences were detected, although a trend was observed (23.53% vs. 12% in HC group). In the RPL group, however, the results reached significance (p = 0.0058), indicating a greater impact in the combined analysis (**Figures 3B**, **4**; **Supplementary Data 3**).

### Analysis of HLA-B -21 dimorphism in patients vs. controls

We propose that the NKG2A receptor may influence NK cell functionality during pregnancy. Our data revealed a significant increase in homozygosity for the TT allele in the uRF group (61%) compared to HC group (30%), with a p-value of 0.0031.

There was also a trend towards increased MM homozygosity in the HC group (11.9%) compared to the uRFgroup (5.9%). However, this difference was not statistically significant (see **Figures 6A**, **4**, and **Supplementary Data 4**).

**Figure 6:**
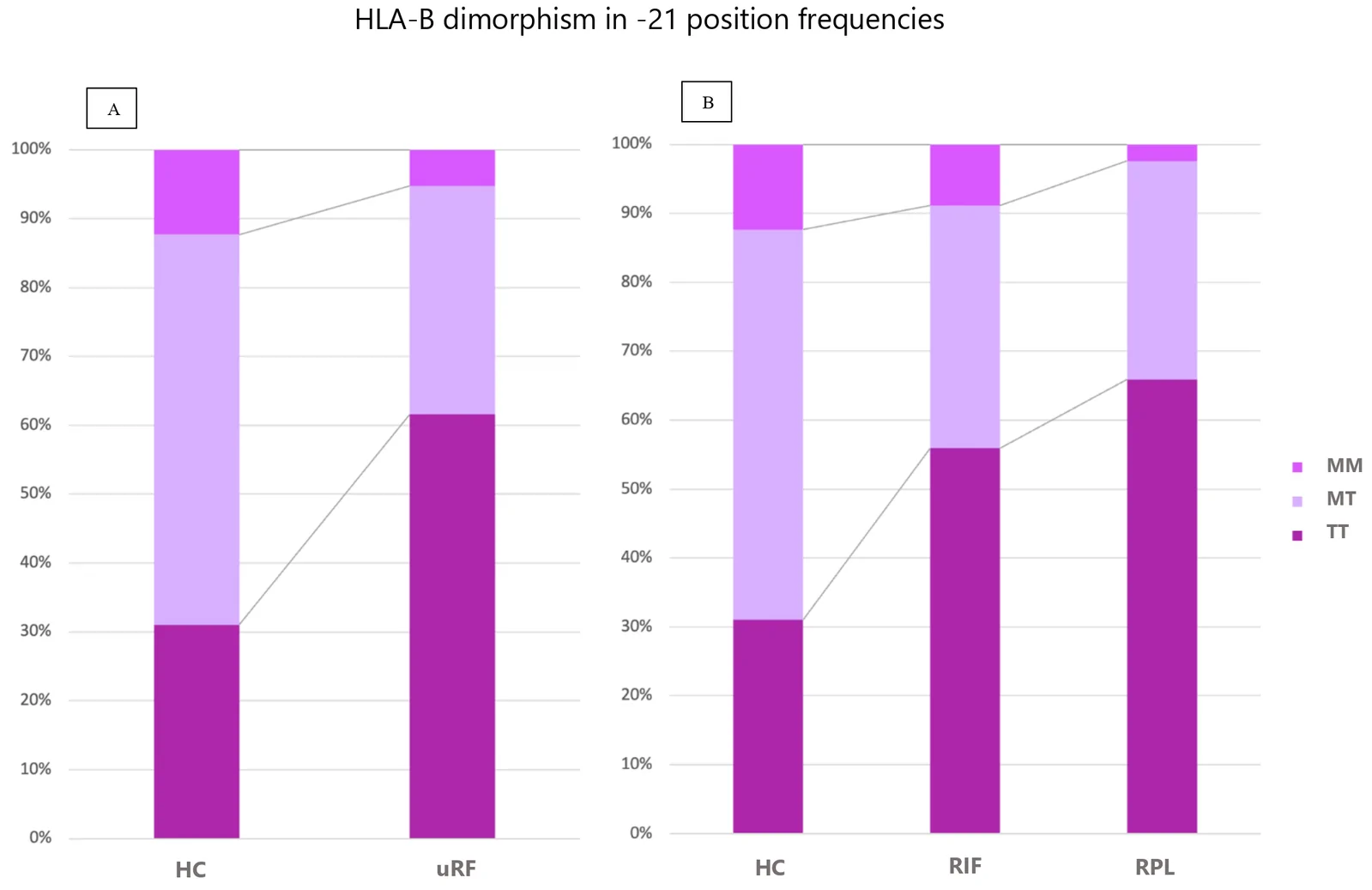
HLA-B dimorphism in -21 position frequencies. **(A)** Frequencies of the B dimorphism at position -21, showing homozygosity for T (TT), homozygosity for M (MM), and heterozygosity (MT) in patients with unexplained reproductive failure (uRF) and women from the healthy control group (HCG). **(B)** Frequency of the B dimorphism at position -21, showing homozygosity for T (TT), homozygosity for M (MM), and heterozygosity (MT) in patients with recurrent implantation failure (RIF) and recurrent pregnancy loss (RPL) in combination with KIR cen AA haplotype and the healthy control group. HC=52 patients, uRF=77 patients, RPL=43 patients, RIF 34= patients.

A higher percentage of HC group had at least one M allele, indicating heterozygosity (54.76% vs. 32.8% in patients, p=0.0252) (see **Figures 6A**, **4**; **Supplementary Data 4**).

As demonstrated in Graph 4B, the RIF and RPL groups exhibited comparable results to the uRF group findings, displaying TT homozygosity at 55.88% and 65.85%, respectively, in contrast to the 30% observed in the control group (p-values of 0.0372 and 0.0025, respectively). Heterozygosity (MT) decreased in both groups (35.29% and 31.7%) compared to the control group (54.76%), with the RPL group showing statistical significance (p=0.0278). While no significant differences in MM homozygosity were identified, a trend towards increased frequency in the HC group was observed (see **Figures 6B**, **4**; **Supplementary Data 4**).

### Matrix combining iKIR missing ligan and -21 HLA-B dimorphism

In this framework we observed that the absence of the KIR2DL1 ligand correlates with a higher presence of the TT dimorphism in the uRF group, at 16% compared to 2.5% in HC group. (**Figure 5A**, **4**; **Supplementary Data 5**).

Since individuals who are homozygous for M are very rare, in this analysis it was decided to combine the MM and MT populations, since the difference is found in individuals who are homozygous for TT.

When analyzing the lack of KIR2DL1 ligands alongside the M dimorphism of HLA-B (refer to individuals that have either M/T or M/), we observed that 42.5% of HC group had this combination, compared to only 12% in the uRF group (p = 0.0004), potentially aiding successful pregnancy development (**Figures 5A**, **4**; **Supplementary Data 4**).

Additionally, the absence of KIR2DL2/3 ligands, a known risk factor for uRF group, increased with the TT dimorphism in the RPL group, reaching 17.3% versus 2.5% in HC group (p = 0.04), suggesting it could serve as a prognostic biomarker for uRF (**Figures 5B**, **4**; **Supplementary Data 4**), this means that TT frequency increases in the absence of C1.

Interestingly, the absence of ligands combined with the M dimorphism did not yield significant differences in either group. Finally, while no significant differences were found when analyzing the absence of KIR3DL1 ligands with TT, the combination with M dimorphism showed a higher presence in HC group (p = 0.03), indicating it may also be beneficial (**Figures 5C**, **4**; **Supplementary Data 4**).

Regarding the results obtained from the RIF and RPL groups, the differences are even more pronounced, especially in the RPL group (**Figures 5D-F**, **4**; **Supplementary Data 5**).

The observed patterns are consistent with those previously identified in the overall uRF group. In the absence of a specific ligand for KIR2DL1, which initially appears advantageous, given its prevalence in the HC group, there is a high prevalence of homozygosity for TT among patients, at 14.7%, compared to 7.31% in the RIF group and 2.5% in the HC group. However, the findings must be considered insignificant due to the small sample size of this stratified group. Research indicates that patients experiencing recurrent reproductive failure are more likely to exhibit homozygosity compared to those lacking the KIR2DL1 ligand (see **Figures 5D**, **4**; **Supplementary Data 5**).

For the MM or MT dimorphism, the frequency is significantly higher in the HC group, presenting at 42.5% compared to 5.88% RIF group and 2.43% in RPL group, with p-values of 0.0023 and 0.0016, respectively (**Figures 5D**, **4**; **Supplementary Data 5**).

When analyzing the KIR2DL2/3 receptor in the absence of its specific ligand C1, which has been determined to be a potential risk marker for recurrent reproductive failure, the data reveals a significantly high frequency in RPl group at 24.4% compared to 2.5% in the HC group. This finding indicates a distinction between the RIF and RPL groups (**Figures 5E**, **4**; **Supplementary Data 5**).

Finally, the results for the KIR3DL1 receptor in the absence of its specific Bw4 ligand are inconclusive. Therefore, we conclude that there is no compelling evidence to analyze KIR3DL1 in combination with the -21HLA-B dimorphism for these study groups. However, there is a tendency for homozygosity for TT in both the RIF and RPL groups, which could yield more conclusive results with a larger sample size (see **Figures 5F**, **4**; **Supplementary Data 5**).

Summarizing, Combining iKIR-ML with −21HLA-B dimorphism categories revealed that absence of the KIR2DL1 ligand co-occurred with TT more often in uRF group(16%) than in HC group (2.5%). Conversely, absence of KIR2DL1 ligand together with an M+ haplotype (MM/MT) was more common in HC group (42.5%) than in uRF group (12%; p=0.0004). Absence of C1 ligands for KIR2DL2/3 combined with TT was enriched in RPL group (17.3% vs 2.5% in controls; p=0.04). KIR3DL1 with missing Bw4 showed no significant associations with TT; the M+ combination was modestly enriched in HC group (p=0.03). (**Figuress 5A–F**, **4**; subgroup sample sizes: RPL n=43; RIF n=34; controls n=75.)

## Discussion

Our results definitively impact the management of uRF. First, we have identified the Cen AA KIR haplotype together with HLA C2 and HLA B −21 T/T. These are risk-associated immunogenetic combinations. They provide a framework for refining risk stratification in reproductive immunology. Women carrying these profiles exhibit a reduced capacity for optimal uNK cell regulation at the maternal–fetal interface. This subgroup is more susceptible to implantation failure or early placental dysfunction. Our results align with prior evidence that maternal KIR AA with fetal/maternal C2 increases the risk of preeclampsia and other placentation disorders, whereas activating KIR2DS1 (typically on KIR B haplotypes) mitigates this risk and associates with higher birth weight (13, 21). Moreover, The association observed between KIR/HLA combinations and reproductive failure is biologically consistent with recent evidence highlighting the pivotal role of uterine NK cells in placental development and pregnancy complications (22).

Second, the integration of iKIR missing ligand patterns (23, 24) into clinical assessment offers an additional layer of predictive value. For example, the absence of C1 ligands for KIR2DL2/3—particularly when combined with the −21 T/T genotype—identifies women with a potentially diminished NKG2A dependent regulatory pathway. NK cells are educated through two main mechanisms: (1) NKG2A+ NK cells interacting with HLA E, and (2) KIR+ NK cells engaging their specific HLA class I epitopes (25, 26). During early pregnancy, uNK cells show an initial upregulation of NKG2A and KIR receptors, followed by a progressive decline in maturation markers. The balance between these two education pathways is strongly influenced by HLA-B–derived peptides that regulate HLA E surface expression and by the presence or absence of key iKIR ligands. This coordinated regulation is essential for shaping uNK cell responsiveness during implantation (21).Our findings differ from those of Faridi et al. (2011) (27), who reported reduced recurrent miscarriage risk in pregnancies where mothers carried KIR2DL1 and both parents were HLA C2/C2. Several methodological and population-related factors may explain this contrast. First, Faridi et al. studied parous women using fetal HLA genotyping, whereas our study evaluates maternal KIR and HLA C independently of paternal haplotype. Second, population-specific KIR/HLA frequencies differ substantially between Northern Indian and Southern European cohorts, which may modulate the impact of inhibitory KIR–C2 interactions. Third, discrepancies between RM vs. RPL definitions and inclusion of biochemical losses may influence associations. These differences highlight that the effect of KIR2DL1–C2 may not be universal but population dependent.

Regarding the NKG2A pathway, Colucci and colleagues reported that individuals carrying the −21 HLA-B T allele, particularly the T/T genotype, exhibit an increased risk—approximately 7%—of insufficient NK-cell education and suboptimal cytokine production (14). Consistent with this, we observed an increased frequency of T/T homozygosity in the uRF group compared with controls. These results parallel previous associations described in preeclampsia and support a model in which reduced HLA-E expression (modulated by the −21B dimorphism rather than by NKG2A itself) may impair uNK functional competence during early gestation.

Third, these immunogenetic markers may contribute to future efforts aimed at improving risk stratification in reproductive medicine. However, the present findings are based on genetic associations and should be considered hypothesis-generating until confirmed in independent prospective studies.

If future studies validate the predictive value of these KIR–HLA combinations, they could eventually contribute to the development of risk-adapted reproductive management strategies. At present, however, there is insufficient evidence to support the use of these genetic markers for clinical decision-making or therapeutic intervention.

Although interventions targeting NK-cell pathways remain experimental, further research will be required to determine whether the immunogenetic profiles identified in this study have any value in predicting response to immunomodulatory approaches.

We integrated the missing-ligand model with the −21 HLA-B dimorphism to investigate a possible synergistic effect, an area that remains poorly explored. Our data show that absence of the KIR2DL1 ligand, combined with the presence of the −21M variant—which was more prevalent in controls—may act as a beneficial biomarker for pregnancy success. This is biologically plausible: KIR2DL1 is the strongest inhibitory KIR, and its engagement with HLA-C2 is known to dampen uNK activation, potentially compromising placentation. Conversely, the −21M allele supports higher HLA-E expression and more robust NKG2A-mediated education, which may counterbalance reduced KIR-mediated inhibition and promote a more physiologic uNK phenotype (14).

Because NKG2A–HLA-E (14) interactions support the acquisition of a tolerant and pro-angiogenic uNK phenotype, future studies should investigate whether individuals carrying these immunogenetic profiles differ in implantation dynamics or placental development.

Similarly, combinations that include preserved −21M-mediated HLA-E expression and absence of the KIR2DL1 ligand were more commonly observed among controls in our cohort. Whether these profiles confer protection requires confirmation in larger independent populations.

Because altered uNK-cell regulation may contribute to abnormal placentation, our findings should also be considered in the broader context of biomarkers associated with impaired fetal growth and pregnancy complications. Recent studies highlight the importance of early biological markers for identifying pregnancies at risk for placental dysfunction and adverse obstetric outcomes (28).

Finally, the combined assessment of Cen/Tel KIR haplotypes, iKIR missing-ligand status, and HLA-B −21 dimorphism provides a framework for exploring the contribution of NK-cell immunogenetics to reproductive failure. Whether these variables can be incorporated into predictive algorithms or clinical tools will require validation in large prospective and multicenter studies.

Importantly, the present study was designed as a genetic association analysis and does not evaluate the clinical performance of the proposed markers, their predictive accuracy, or their utility in guiding patient management. Therefore, all potential clinical implications discussed here should be regarded as exploratory and hypothesis-generating. Independent validation in larger prospective cohorts will be necessary before considering any implementation in reproductive counseling, risk assessment, or therapeutic decision-making.

### Limitations

A major limitation of the present study is the absence of functional validation. Although HLA-B −21 polymorphism and inhibitory KIR-ligand combinations are known to influence NK-cell education, genotype does not necessarily predict receptor expression, HLA-E surface density, decidual NK-cell phenotype, or *in vivo* cellular function. Therefore, the biological mechanisms proposed here should be considered hypothesis-generating and require validation through protein-expression studies, flow-cytometric characterization of decidual NK-cell subsets, and functional assays evaluating cytokine production and trophoblast interactions.

Also an important limitation of this study is the number of genetic comparisons performed relative to the cohort size. Although correction for multiple testing was applied to predefined primary analyses, several exploratory comparisons were evaluated using nominal p-values. Consequently, some associations may represent false-positive findings and should therefore be considered hypothesis-generating until validated in larger independent cohorts.

The relatively modest sample size, particularly within the stratified RPL and RIF analyses, limits statistical power and may have prevented detection of additional significant associations. Replication in larger multicenter cohorts will be necessary to confirm the predictive value of the proposed biomarkers.

## Data Availability

The datasets presented in this study can be found in online repositories. The names of the repository/repositories and accession number(s) can be found in the article/**Supplementary Material**.
