# Supplementary material for: Combination of iKIR missing ligand hypothesis and HLA-B T/T genotypes as predictive biomarkers for recurrent pregnancy loss: a novel algorithmic approach
Source: Front Immunol. 2026 Oct 8;17:1866398. doi: 10.3389/fimmu.2026.1866398 (PMC13691304; doi:10.3389/fimmu.2026.1866398)
Supplement: Supplementary file 1 [file Table1.docx]

|  | **HC-Group (N=75)** | | **uRF-Group (N=77)** | |  |  |  |
| --- | --- | --- | --- | --- | --- | --- | --- |
|  | **N** | **%** | **N** | **%** | **Odds Ratio** | **95% CI** | **P-Value** |
| Classical classification | | | | | | | |
| **KIR AA** | 15 | 20.00 | 32 | 41.02 | 2.782 | 1.3495 to 5.7377 | **.0056** |
| **KIR AB** | 60 | 80.00 | 45 | 59.98 | 0.3409 | 0.1655 to 0.7021 | .0035 |
| **KIR BB** | 3 | 4.00 | 0 | 0.00 | 0.1319 | 0.0067 to 2.5986 | .1829 |
| Centromeric and Telomeric classification | | | | | | | |
| **CenAA** | 26 | 34.67 | 44 | 56.41 | 2.438 | 1.2694 to 4.6860 | **.0075** |
| **CenAB** | 35 | 46.67 | 28 | 35.89 | 0.64 | 0.3348 to 1.2234 | .1770 |
| **CenBB** | 14 | 18.67 | 5 | 7.60 | 0.3512 | 0.1271 to 0.9705 | **.0436** |
| **TelAA** | 42 | 56 | 55 | 70.51 | 18.789 | 0.9644 to 3.6604 | .0638 |
| **TelAB** | 27 | 36 | 22 | 28.21 | 0.6984 | 0.3530 to 1.3818 | .3025 |
| **TelBB** | 6 | 8 | 1 | 1.28 | 0.1494 | 0.0175 to 1.2717 | .0819 |

**Supplementary data 1.**  Frequencies of classical KIR genotypes in unexplained recurrent failure (uRF), recurrent reproductive failure (RPL) and the healthy control group (HC). KIR: killer immunoglobulin-like receptor of NK cells; RPL: recurrent reproductive failure.

| Classic classification | | | | |
| --- | --- | --- | --- | --- |
|  |  | **KIR AA** | **KIR AB** | **KIR BB** |
| HC (N=75) | **N** | 15 | 60 | 3 |
|  | **%** | 20.00 | 80.00 | 4.00 |
| RIF-group (N=34) | **N** | 16 | 18 | 0 |
|  | **%** | 47.05 | 52.94 | 0.00 |
|  | **Odds Ratio** | 3,5556 | 0.2813 | - |
|  | **CI 95%** | 1.4754 - 8.5685 | 0.1167 to 0.6778 | - |
|  | P-value | **.0047** | **.0047** | - |
| RPL-group (N=43) | **N** | 20 | 21 | 0 |
|  | **%** | 48.78 | 51.22 | 0 |
|  | **Odds Ratio** | 3,8095 | 0.2625 | - |
|  | **95% CI** | 1.6549 - 8.7693 | 0.1140 - 0.6043 | - |
|  | P-value | **.0017** | **.0017** | - |

| Centromeric and telomeric KIR genotype classification | | | | | | | |
| --- | --- | --- | --- | --- | --- | --- | --- |
|  | | **CenAA** | **CenAB** | **CenBB** | **TelAA** | **TelAB** | **TelBB** |
| HC (N=75) | **N** | 26 | 35 | 14 | 42 | 27 | 6 |
|  | **%** | 34.67 | 46.67 | 18.67 | 56 | 36 | 8 |
| RIF-group (N=34) | **N** | 19 | 14 | 3 | 26 | 10 | 1 |
|  | **%** | 55.88 | 41.17 | 8.82 | 76.47 | 29.41 | 2.94 |
|  | **Odds Ratio** | 2,3872 | 0.8000 | 0.4217 | 2,5536 | 0.7407 | 0.3485 |
|  | **CI 95%** | 1.0437 - 5.4599 | 0.3524 - 1.8163 | 0.1127 - 1.5781 | 1.0235 - 6.3708 | 0.3086 - 1.7779 | 0.0403 - 3.0136 |
|  | P-value | **.0393** | 0.5937 | 0.1997 | **.0444** | .5017 | .3382 |
| RPL-group (N=43) | **N** | 24 | 20 | 3 | 34 | 15 | 3 |
|  | **%** | 58.53 | 48.78 | 7.31 | 82.92 | 36.58 | 7.31 |
|  | **Odds Ratio** | 3,0154 | 1,0884 | 0.3440 | 3,8163 | 1,0256 | 0.9079 |
|  | **95% CI** | 1.3529 - 6.7206 | 0.5080 - 2.3323 | 0.0927 - 1.2763 | 1.5019 - 9.6973 | 0.4649 - 2.2629 | 0.2148 - 3.8372 |
|  | P-value | .**0070** | .8275 | .1106 | **.0049** | .9500 | .8955 |

**Supplementary data 2.** Frequency of HLA-C haplotypes in combination with centromeric KIR genotype in unexplained recurrent failure (uRF), recurrent reproductive failure (RPL) and the healthy control group (HC); OR: odds ratio; CI: confidence interval. uRF: unexplained recurrent failure, RPL: recurrent reproductive failure and RIF: recurrent implantation failure.

| **Combinación de haplotipo HLA-C con KIR centromérico** | | | | | | | |
| --- | --- | --- | --- | --- | --- | --- | --- |
|  |  | **Cen AA** | | | **Cen BB** | | |
|  |  | **C1C1** | **C1C2** | **C2C2** | **C1C1** | **C1C2** | **C2C2** |
| **HC** | **N** | 17 | 6 | 3 | 4 | 6 | 3 |
|  | **%** | 65,38 | 23,07 | 11,5 | 30,76 | 46,15 | 23,07 |
| **uRF** | **N** | 12 | 19 | 3 | 1 | 4 | 1 |
|  | **%** | 30.00 | 47.50 | 22.50 | 16.66 | 66.66 | 16.66 |
|  | **Odds Ratio** | 0.2269 | 30159 | 22258 | 0.4500 | 23333 | 0.6667 |
|  | **IC 95%** | 0.0791 to 0.6507 | 1.0005 to 9.0909 | 0.5415 to 9.1499 | 0.0389 to 5.2091 | 0.3103 to 17.5453 | 0.0545 to 8.1617 |
|  | **P-Value** | .0058 | .0499 | .2673 | .5228 | .4104 | .7510 |
| **RIF** | **N** | 7 | 12 | 3 | 0 | 0 | 3 |
|  | **%** | 36,84 | 63,15 | 15,78 | 0 | 0 | 15,78 |
|  | **Odds Ratio** | 0.1373 | 5,7143 | 1,4375 | - | - | 3,6667 |
|  | **95% CI** | 0.0431 - 0.4375 | 1.5506 - 21.0584 | 0.2567 - 8.0506 | - | - | 0.4734 - 28.4013 |
|  | **P-Value** | **.0008** | **.0088** | .6797 | - | - | .2135 |
| **RPL** | **N** | 4 | 9 | 9 | 0 | 3 | 0 |
|  | **%** | 16,66 | 37,5 | 37,5 | 0 | 12,5 | 0 |
|  | **Odds Ratio** | 0.1059 | 2 | 46000 | - | 13333 | - |
|  | **95%CI** | 0.0276 - 0.4058 | 0.5840 to 6.8491 | 1.0687 - 19.8000 | - | 0.1957 - 9.0831 | - |
|  | **P-Value** | **.0011** | 0.2697 | **.0404** | - | .7689 | - |

**Suplementary data 3.** Frequency of HLA-C2, C1, and Bw4 haplotypes specific to the KIR2DL1, KIR2DL2/3, and KIR3DL1 receptors, respectively, in unexplained recurrent failure (uRF), recurrent reproductive failure (RPL) and the healthy control group (HC); OR: odds ratio; CI: confidence interval. uRF: unexplained recurrent failure; RIF: recurrent implantation failure; RPL: recurrent reproductive failure.

|  |  | **2DL1 (C2 absent)** | **2DL2/2DL3 (C1 absent)** | **3DL1 (Bw4 absent)** |
| --- | --- | --- | --- | --- |
| **HC** | **N** | 34 | 9 | 9 |
|  | **%** | 45.94 | 12 | 12 |
| **uRF** | **N** | 21 | 22 | 28 |
|  | **%** | 26.9 | 28.2 | 35.9 |
|  | **Odds Ratio** | 0.4443 | 2.881 | 1.792 |
|  | **95% CI** | 0.2260 to 0.8735 | 1.2272 to 6.7633 | 0.7679 to 4.1821 |
|  | **P-Value** | .0187 | .0151 | .1773 |
| **RIF** | **N** | 11 | 8 | 12 |
|  | **%** | 32.35 | 23.53 | 35.29 |
|  | **Odds Ratio** | 0.5767 | 22.564 | 35.455 |
|  | **95% CI** | 0.2464 - 1.3497 | 0.7857 - 6.4803 | 1.3460 - 9.3388 |
|  | **P-Value** | .2045 | .1306 | .0104 |
| **RPL** | **N** | 9 | 14 | 16 |
|  | **%** | 23.95 | 34.14 | 39.02 |
|  | **Odds Ratio** | 0.3392 | 38.025 | 41.600 |
|  | **95% CI** | 0.1424 - 0.8080 | 1.4712 - 9.8281 | 1.6663 - 10.3855 |
|  | **P-Value** | **.0146** | **.0058** | **.0023** |

**Supplementary data 4** Comparison of the frequency of -21 HLA-B dismorphism, grouped in TT (homocygous), MT (heterocygous) and MM (homocygous) in unexplained recurrent failure (uRF), recurrent reproductive failure (RPL) and the healthy control group (HC).

|  |  | **TT** | **MT** | **MM** |
| --- | --- | --- | --- | --- |
| **HC** | **N** | 16 | 28 | 7 |
|  | **%** | 30 | 54.76 | 11.9 |
| **uRF** | **N** | 45 | 25 | 4 |
|  | **%** | 61 | 32.8 | 5.2 |
|  | **Odds Ratio** | 33.462 | 0.4130 | 0.4169 |
|  | **95% CI** | 1.5022 to 7.4535 | 0.1904 to 0.8960 | 0.1056 to 1.6465 |
|  | **P-Value** | .0031 | .0252 | .2119 |
| **RIF** | **N** | 19 | 12 | 3 |
|  | **%** | 55,88 | 35,29 | 8,82 |
|  | **Odds Ratio** | 27282 | 0.4269 | 0.8903 |
|  | **95% CI** | 1.0615 to 7.0117 | 0.1675 to 1.0879 | 0.1982 to 3.9985 |
|  | **P-Value** | **.0372** | .0745 | .8795 |
| **RPL** | **N** | 27 | 13 | 1 |
|  | **%** | 65,85 | 31,7 | 2,43 |
|  | **Odds Ratio** | 41538 | 0.3634 | 0.2300 |
|  | **95% CI** | 1.6527 to 10.4401 | 0.1475 to 0.8953 | 0.0258 to 2.0520 |
|  | **P-Value** | **.0025** | **.0278** | .1881 |

**Supplementary data 5**. Matrix of the frequency frequency of -21 HLA-B dismorphism, grouped in TT (homocygous), MT (heterocygous) and MM (homocygous) with frequency of iKIR missing ligand hypothesis in unexplained recurrent failure (uRF), recurrent reproductive failure (RPL) and the healthy control group (HC)

|  |  | **2DL1** | **2DL1** | **2DL2/3** | **2DL2/3** | **3DL1** | **3DL1** |
| --- | --- | --- | --- | --- | --- | --- | --- |
|  |  | **(C2 absent)/TT** | **(C2 absent)/M** | **(C1 absent)/TT** | **(C1 absent)/M** | **(Bw4 absent)/TT** | **(Bw4 absent)/M** |
| **HC-group** | **N** | 12 | 10 | 13 | 9 | 8 | 0 |
|  | **%** | 16 | 12 | 17.3 | 12 | 10.67 | 0 |
| **uRF-group** | **N** | 1 | 17 | 1 | 2 | 0 | 5 |
|  | **%** | 2.5 | 42.5 | 2.5 | 2.5 | 0 | 12.5 |
|  | **Odds Ratio** | 7.55 | 0.185 | 8.31 | 2.59 | - | - |
|  | **95% CI** | 0.9440 to 60.3598 | 0.0723 to 0.4709 | 1.0453 to 66.0849 | 0.5319 to 12.6208 | - | - |
|  | **P-value** | **.05** | **.0004** | **.04** | .23 | .11 | **.03** |
| **RIF-group** | **N** | 5 | 2 | 3 | 5 | 5 | 8 |
|  | **%** | 14.7 | 5.88 | 8.82 | 14.7 | 14.7 | 23.53 |
|  | **Odds Ratio** | 6,8966 | 0.0882 | 3,871 | 33621 | 15,4746 | 2,2154 |
|  | **95% CI** | 0.7644 to 62.2193 | 0.0186 to 0.4190 | 0.3837 to 39.0484 | 0.6088 to 18.5670 | 0.8235 to 290.7708 | 0.6502 to 7.5488 |
|  | **P-value** | .0853 | **.0023** | .2511 | .1643 | .0672 | .2035 |
| **RPL-group** | **N** | 3 | 1 | 10 | 2 | 7 | 8 |
|  | **%** | 7.31 | 2.43 | 24.4 | 4.87 | 17.07 | 19.51 |
|  | **Odds Ratio** | 3.000 | 0.0353 | 129032 | 1000 | 18,0435 | 1,7455 |
|  | **95% CI** | 0.2992 to 30.0803 | 0.0044 to 0.2823 | 1.5667 to 106.2681 | 0.1340 to 7.4605 | 0.9946 to 327.3220 | 0.5189 to 5.8716 |
|  | **P-value** | .3503 | **.0016** | **.0174** | 1.000 | .0504 | .3681 |
